# Supplementary material for: Programmable black phosphorus image sensor for broadband optoelectronic edge computing
Source: Nat Commun. 2022 Mar 18;13:1485. doi: 10.1038/s41467-022-29171-1 (PMC8933397; doi:10.1038/s41467-022-29171-1)
Supplement: Supplementary file 1 — Supplementary Information [file 41467_2022_29171_MOESM1_ESM.pdf]

SUPPLEMENTARY INFORMATION

Programmable black phosphorus image sensor for broadband optoelectronic edge computing

Seokhyeong Lee[1†], Ruoming Peng[1†*], Changming Wu[1], and Mo Li[*,2*]

[1]*Department of Electrical and Computer Engineering, University of Washington, Seattle, WA 98195, USA*

[2]*Department of Physics, University of Washington, Seattle, WA 98195, USA*

[*] Corresponding author: pruoming@uw.edu, moli96@uw.edu

[†] These authors contributed equally to this work.

Supplementary Note 1. With the electrostatic tunability of 2D materials, many researchers have demonstrated electrically/optically programmable conductance/persistent-photoconductance (PPC) using varied materials for electrical channels and charge storage layers as summarized in Supplementary Table 1. On top of PPC, the programmable photoresponsivity (R) of the bP-PPT device enables instantaneous in-sensor image processing. The multifunctional operation in both optical and electrical domain together with the array geometry realizes CNN to recognize handwritten digits with 92% accuracy. The optical programmability sets over 5-bit of stable states and suggests parallel and remote programmability.

Supplementary Table 1. Comparison of key features and performance of our device with prior works.

Ref	Material		Programming method	Physical Attribute		CNN Accuracy	# of stable states
	Channel	Storage		Weight	Input		
**This work**	bP	AHA	Electrical & Visible light	g, R	E, O	92	36
1	bP	POx	UV light	g	E	90	*N.D.*
2	WSe_2	h-BN	Electrical	g, R	E, O	90	*N.D.*
3	MoS_2	AlOx	Electrical & Visible light	g	E	94	4
4	bP	AHA	Electrical	g	E	*N.D.*	2
5	bP	cPVP	Electrical	g	E	*N.D.*	5
6	MoS_2	graphene	Electrical	g	E	*N.D.*	*N.D.*
7	InSe	graphene	Electrical	g	E	*N.D.*	16
8	WSe_2	No memory	Electrical	R	O	99	*N.D.*

* Input E: electrical, O: optical

* Weight: g for conductance, R for responsivity

* *N.D.*: Not demonstrated

* AHA: $Al_2O_3/HfO_2/\ Al_2O_3$ stack, cPVP: cross-linked poly-4-vinylphenol

Supplementary Note 2. The retention time of the bP-PPT device is estimated by fitting the drain-source current I_{ds} versus time curves with the power function[9,10], $f(t) = At^k$ (Supplementary Figure 1a). The black and red curves indicate the highest conductive state programmed by the positive electrical pulse and the lowest conductive state set by the negative pulse, respectively. 30 % retention time is estimated to be about 1 year.

Supplementary Figure 1. The retention time and endurance performance of a bP-PPT device. (a) Time trace of state retention for two conductive states. (b) 3 bits (8 states) programming with electrical pulses. The first 18 V voltage pulse sets the device to the highest conductance state. Following depressive pulses of 20-ms with different voltage from -4 V to -12 V set the device to 8 different states (color coded). (c) Endurance test of the bP-PPT devices for 200 cycles of repeated procedure described in (b).

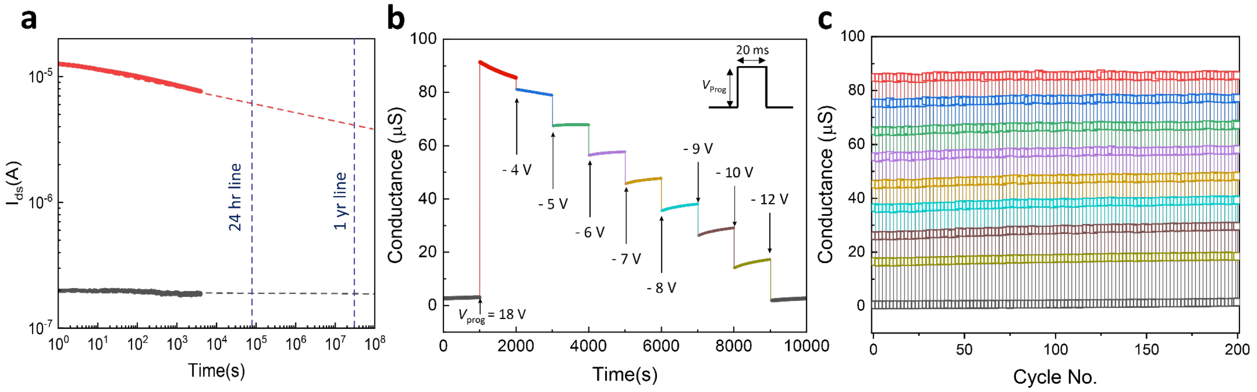

Supplementary Note 3. The charge stored in the dielectric layer (σ_s) (Supplementary Figure 2a) is modulated by the maximum (minimum) voltage (V_{max}) applied on the top gate during a voltage sweep. Supplementary Figure 2b shows that the hysteresis window increases with the larger maximum voltage, indicating a larger number of stored charges. The stored charge density Δn_s can be estimated with the following expression:[3]

$$\Delta n_s(V_{max}) = \frac{\sigma_s}{q} = \frac{\Delta V_{th}\epsilon_1}{d_1 q} = \frac{\Delta V_{th}(V_{max})C_{bl}}{q}$$

, where $\Delta V_{th}(V_{max})$ is the threshold voltage shift toward the positive voltage direction when V_{max} is changed, C_{bl} is the specific capacitance of the blocking layer, q is the electron charge, and ϵ_1 is the permittivity of the Al_2O_3 blocking layer. The maximal charge density that can be stored in the HfO_2 layer is estimated to be $\Delta n_{s,max} = 1.8 \times 10^{13}\ cm^{-2}$, for electrons or holes. In addition, as it will be further discussed in Supplementary Note 4 and 5 with a theoretical model of bP-PPT, the charge density stored in the storage layer is determined by V_{max} when the gate sweeping takes longer than 100 ms. Thus, the charge density stored in the storage layer is related to the change of threshold voltage which determines the conductance of bP-PPT channel at zero gate voltage as shown in Supplementary Figure 2c.

Supplementary Figure 2. Charge density in the AHA stack of bP-PPT. (a) The schematics of electric field and charge distribution when gate voltage across the dielectric stack is applied. (b) the hysteresis loop for gate voltage swept with different maximum voltage. (c) The conductance of bP-channel without gate voltage versus the threshold voltage shift and the charge density stored in the storage layer. Approximately linear relation between the n_{stored} and the conductance of bP-channel will be used for further analysis of optical programming in Supplementary Note 6.

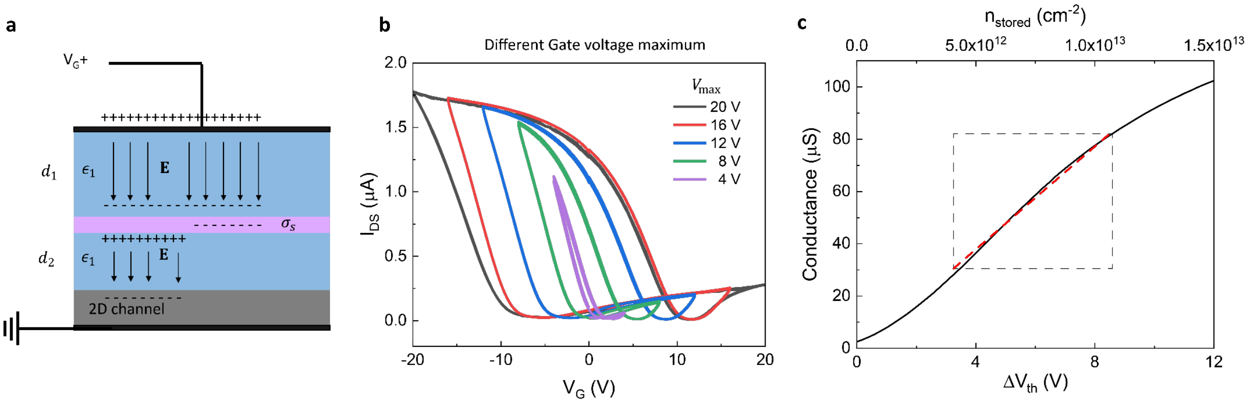

Supplementary Figure 3. Plot for the photocurrent versus the incident optical power. The photocurrent linearly depends on the optical power dependence when the bP-PPT device is programmed to the low-g and high-g states with photoresponsivity of 60 mA/W and 20 mA/W, respectively. The programmable photoresponsivity (R) enables the linear calculation by measuring the photocurrent: $I_{Ph} = R \cdot P_{NIR}$. The bias voltage is set constant at 50 mV.

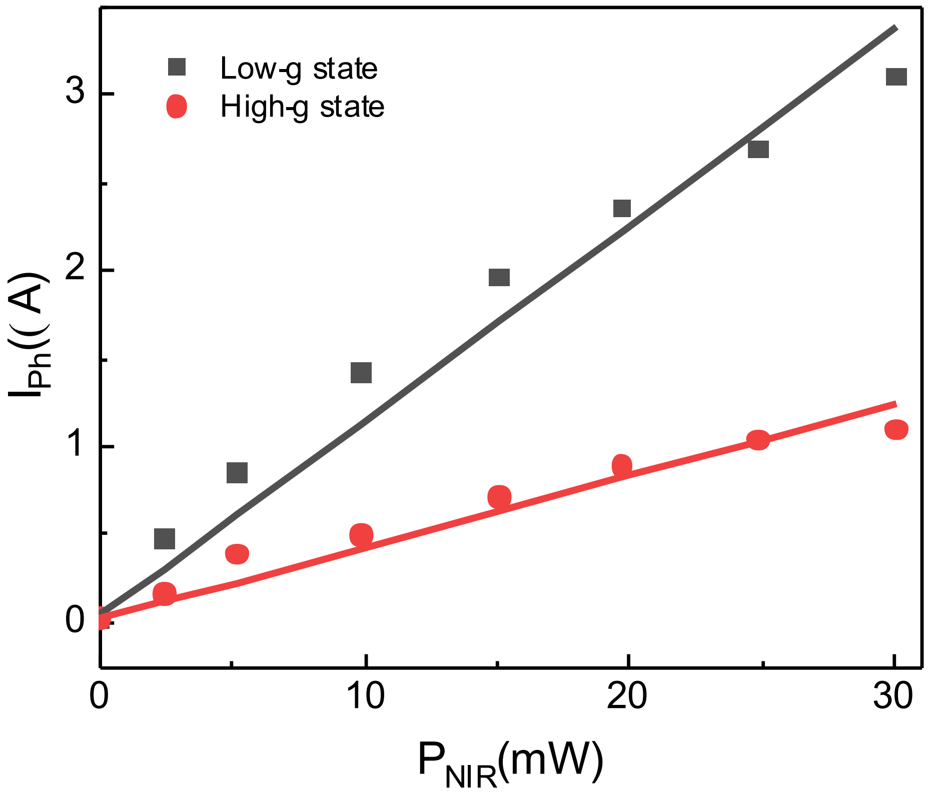

Supplementary Figure 4. States of different conductance and photoresponsivity. (a) The source-drain current $I_{ds}(= I_{dark} + I_{ph})$ measured with/without optical illumination at 100 mV bias voltage. (b) The photocurrent of different states. The higher the conductive states, the lower photoresponsivity is measured, consistent with the reference [11]. The state numbered as S2 is equivalent to #1 in Fig. 2e of the main text (S3 to #22, S4 to #35). S1, S5, and S6 are obtained with gate voltage keeping on (not optically programmable states). The telecom band laser is used at 1550-nm wavelength with 10-mW power.

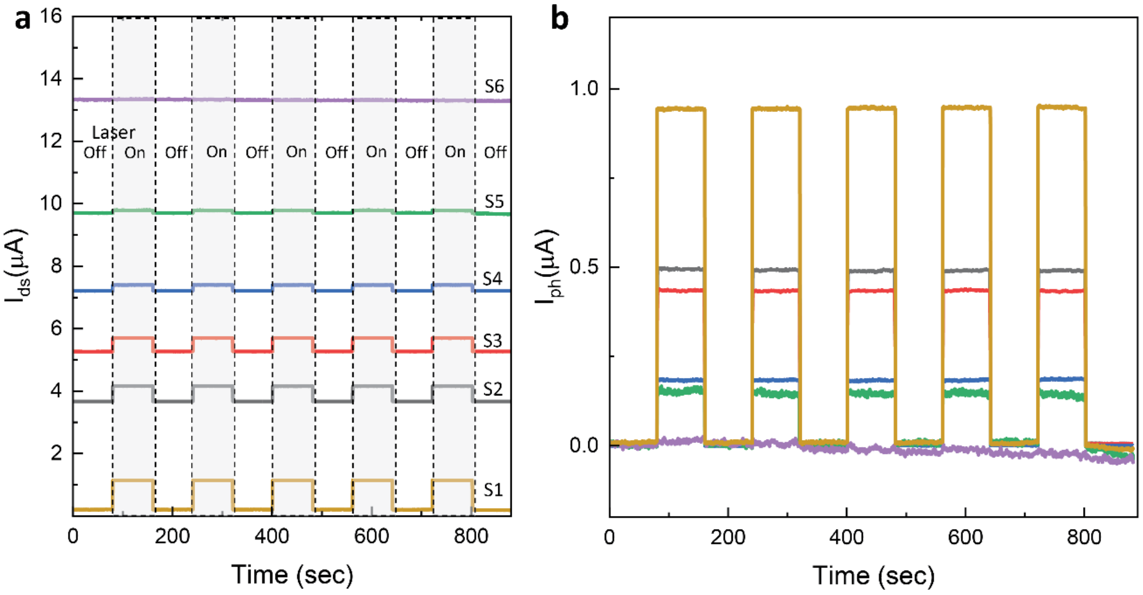

Supplementary Figure 5. Pulse energy for setting different conductance states. (a) The pulse energy for each pulse number. (b) The conductance of each state (the same as Fig. 2e in the main text). The pulse number 1 changes the state from #0 to #1, and the pulse number 2 changes the state from #1 to #2, and so on. The required pulse energy for state-change grows exponentially because the reduced charge density in the storage layer also reduces the built-in electric field that assists the escape of photoexcited charges from the storage layer to the bP-channel, which is further discussed in Supplementary Note 4, 5 and 6. Inset: the time stability of the three representative states with more than 1000 sec of retention. (c) The statistics for each state. The conductance states of our bP-PPT devices are differentiated with the following two criteria:

(1) Each state is stable within a 99 % confidence interval (C.I.) (2.5 standard deviations) upon the repeated setting of the desired states.
(2) The separation between the states is more than 6 times of the standard deviation (6σ) so that each state can be clearly distinguished from the two adjacent states.

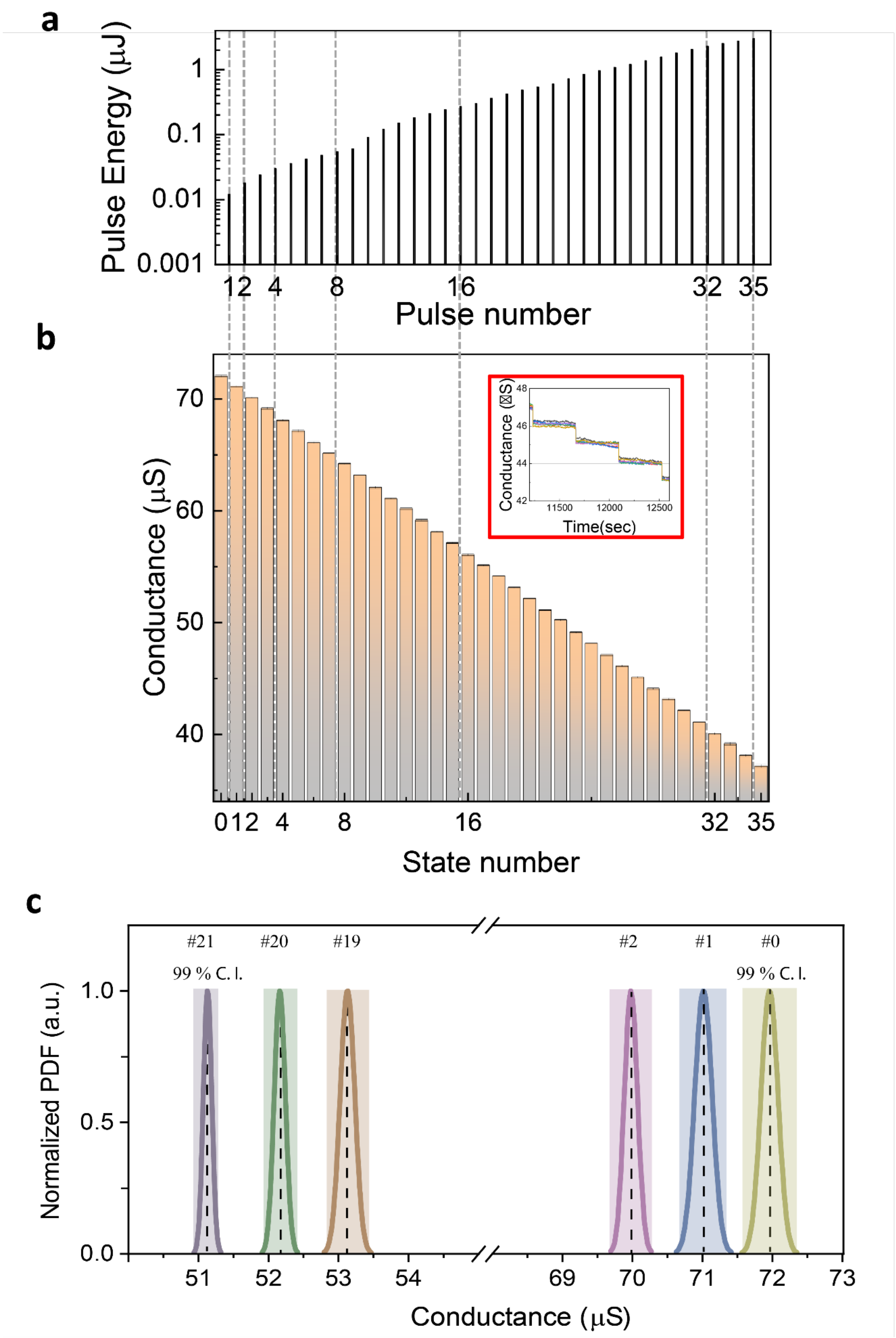

a
Pulse Energy (μJ)
1
0.1
0.01
0.001
1 2 4 8 16 32 35
Pulse number
b
Conductance (μS)
70
60
50
40
0 1 2 4 8 16 32 35
State number
Conductance (⊠S)
48
46
44
42
11500
12000
12500
Time(sec)
c
#21
#20
#19
#2
#1
#0
99 % C. I.
99 % C. I.
Normalized PDF (a.u.)
1.0
0.5
0.0
51 52 53 54 69 70 71 72 73
Conductance (μS)

Supplementary Note 4. The working principle of the bP-PPT device can be modeled by the Fowler-Nordheim tunneling (FN tunneling), which explains the charge trapping and de-trapping mechanism by electrical and optical control.

Supplementary Figure 6a depicts the band alignment of the bP channel, Al_2O_3 tunnel layer, and HfO_2 charge storage layer. When a sufficiently large voltage is applied to the top ITO gate (Fig. 1e in the main text), the large electric field across the tunneling layer can lead to the FN tunneling. A tunneling current will be injected into the HfO_2 trapping layer, where charges are trapped at trapping sites with energy in the bandgap[12]. After the applied gate voltage is removed, these metastable trapped charges remain in the HfO_2 layer and induce effective gating to the bP channel, modulating its optical and electric properties. Supplementary Figure 6b illustrates the band diagram under the built-in electric field by these trapped charges.

Under optical illumination, the trapped electrons can be excited and escape from the trap site to tunnel back to the bP-channel, facilitated by the built-in field as in Supplementary Figure 6b. The amount of tunneling charges can be precisely controlled by the optical power and pulse duration to realize 36 intermediate states. The electric field distributions with and without gate voltage are depicted in Supplementary Figure 6c and d, for different charge densities in the charge storage layer (long-dashed line, short-dashed, and straight lines for the highest, moderate, and no charges, respectively). To estimate the electrical programming/erasing speed and optical programming speed, we calculate the FN tunneling current with a triangle barrier of bP/AHA/ITO device. The quantum tunneling transmission function TC(ξ) is first calculated using the Wentzel-Kramers-Brillouin (WKB) approximation, and is given by [6,13],

$$TC(\xi) = \exp\left(-\frac{2}{\hbar}\int_0^{t_{ox}} \sqrt{2m_{ox}(V(x)-\xi)}\,dx\right) \tag{1}$$

where t_{ox} is the thickness of the tunneling layer (Al_2O_3), m_{ox} is the effective mass of the carriers in the tunneling layer, $V(x)$ is the potential function of the triangle-barrier, and ξ is the energy of incident carriers referenced to the Fermi energy of the electrode.

When the gate voltage is applied, the electric field across the tunneling Al_2O_3 layer is calculated using the capacitor model:

$$V_G = \frac{\sigma_1}{\epsilon_{AlO}} t_{blc} + \frac{\sigma_1}{\epsilon_{HfO_2}}(t'_{str}) + \frac{\sigma_1 + \sigma_{HfO_2}}{\epsilon_{Hfo}}(t_{Hfo} - t'_{str}) + \frac{\sigma_1 + \sigma_{HfO_2}}{\epsilon_{AlO}} t_{tnl} \quad (2)$$

$$E_{ox} = \frac{V_{tnl}}{t_{tnl}} = \frac{\sigma_1 + \sigma_{HfO_2}}{\epsilon_{AlO}} \quad (3)$$

where σ_1, σ_{HfO_2} are the charge density at the interface between the gate electrode and the blocking layer, and the charge density stored in the HfO_2 layer, ϵ_{AlO}, ϵ_{HfO} are the dielectric constant for Al_2O_3 and HfO_2, t_{tnl}, t_{HfO}, t_{blc} and t'_{str} are the thickness of tunneling layer, charge storage layer, blocking layer, and the position for the barycenter of the stored charge, respectively. We assume the charge density is uniformly spread in the HfO_2 layer so that the barycenter is assumed as the center of the HfO_2 layer. Accordingly, the tunneling current through the tunneling layer can be estimated as [13]:

$$J_{FN} = \frac{m_e q^3}{8\pi m_{ox} h q \Phi_B} E_{ox}^2 \exp\left(-\frac{4\sqrt{2m_{ox}}}{3\hbar q E_{ox}} (q\Phi_B)^{\frac{3}{2}}\right) \quad (4)$$

where q, h, m_e, and Φ_B are electron charge, Planck's constant, electron mass, potential energy barrier at the bP and Al_2O_3 interface, respectively.

The stored charge density in the HfO_2 layer ($\sigma_{HfO_2}(t)$) (negative for electrons and positive for holes) is the integration of the tunneling current density over the operation time t,

$$\sigma_{HfO_2}(t) = \int_0^t J_{FN}(t')dt'. \quad (5)$$

The stored charge density after the programming by gate pulse can induce the effective gating to the bP-channel. Meanwhile, those stored charges in the HfO_2 layer can form the built-in electric field across the tunneling layer and cause the charges leakage to the bP channel. The tunneling

current of the leakage process depends on the tunneling coefficient and the carrier density regarding the process, which can be described by Tsu-Esaki formula[14]:

$$J_{leak} = -q\frac{dn_{stored}}{dt} = \frac{q}{4\pi^3\hbar}\int_{\xi_{min}}^{\xi_{max}} TC(\xi)g(\xi)f(\xi)\, d\xi \tag{6}$$

where $g(\xi)$ and $f(\xi)$ are the density of state of charge carriers and distribution function, respectively. For simplicity, we assume charge trapping in HfO_2 is dominated by one type of traps so simplify $g(\xi)$ with delta function, $g(\xi) = n_{stored}\delta(\xi - \xi_{trap})$. $f(\xi)$ is Fermi-Dirac distribution function. Then, since the tunneling coefficient depends on the built-in field and the stored charge density, we have:

$$-\frac{\mathrm{d}n_{stored}}{dt} \propto \left\{ \begin{matrix} TC\left(\xi_{trap}, E_{built}(n_{stored})\right) * n_{stored} + TC\left(\xi_{th}, E_{built}(n_{stored})\right) * n_{th} \\ +TC\left(\xi_{opt}, E_{built}(n_{stored})\right) * n_{opt} \end{matrix} \right\} \tag{7}$$

where the first and second terms of the right-hand side are related to the leakage process without any external illumination. The first is the direct tunneling due to built-in field from the traps of the stored charges and the second term is thermally excited charges from traps to conduction band with energy ξ_{th} and contributes to tunneling. In the third term, we also consider the optical excitation of the stored charges with the photon energy $\hbar\omega$ and the excited electron energy $\xi_{opt} = \hbar\omega + \xi_{trap}$, which are related to the tunneling coefficient TC, while the stored charge density in the storage layer also affects the built-in field that assists the tunneling process.

Supplementary Figure 6. The working principle of programming and erasing of bP-PPT device. (a) Band diagram of bP/AlO/HfO layer when 18 V top gate voltage is applied. Electrons from bP tunnel into HfO_2 layer and are trapped below the conduction level. (b) Band diagram of AHA charge storage layer with a charge density of $1.5 \times 10^{13}\ \mathrm{cm}^{-2}$ in the HfO_2 layer without top gate voltage. Trapped charges can optically be excited and removed from the charge storage layer. (c), (d) The band alignment changes with the trapped charged density with (c) and without (d) the top gate applied. Long-dashed line, short-dashed line, and straight line refer to charge density of 1.5, 0.5, and 0 ($\times\ 10^{13}\ \mathrm{cm}^{-}2$), respectively.

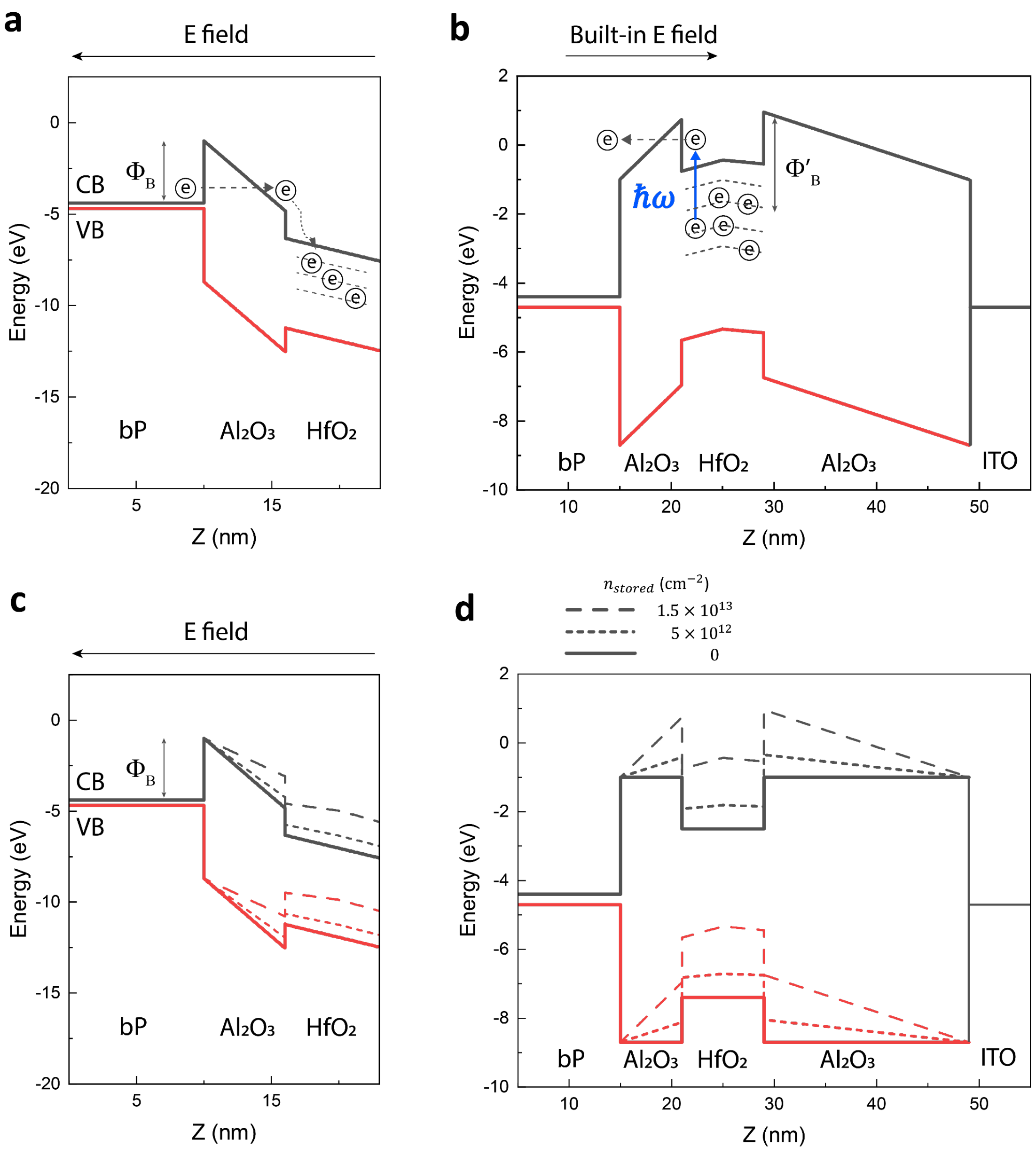

Supplementary Note 5. From equations (3), (4), and (5) in S.I. Note 4, the stored charges can screen the effective electric field in the tunneling layer. Hence, the FN tunneling current is suppressed with increasing density of stored charge, which we calculate and plot in Supplementary Figure 7a. The initial programing of conductance of the bP-PPT channel requires 10s of ms gate pulse to saturate the charge density in the trapping layer as shown in Fig. S7b. Different gate voltages and pulse time are considered in Supplementary Figure 7 a and b, which result in the different conductive states as shown in Fig. 2C of the main text. The shorter pulses on the order of μs can be used to program the devices in smaller steps of conductance.

Supplementary Figure 7. The electrical programming speed. (a) The FN tunneling versus the trapped charge density in the storage layer with different top gate voltage V_G. (b) Different charge densities in the storage layers depending on the pulse duration. Inset: Zoomed-in plot of (b) with shorter pulses.

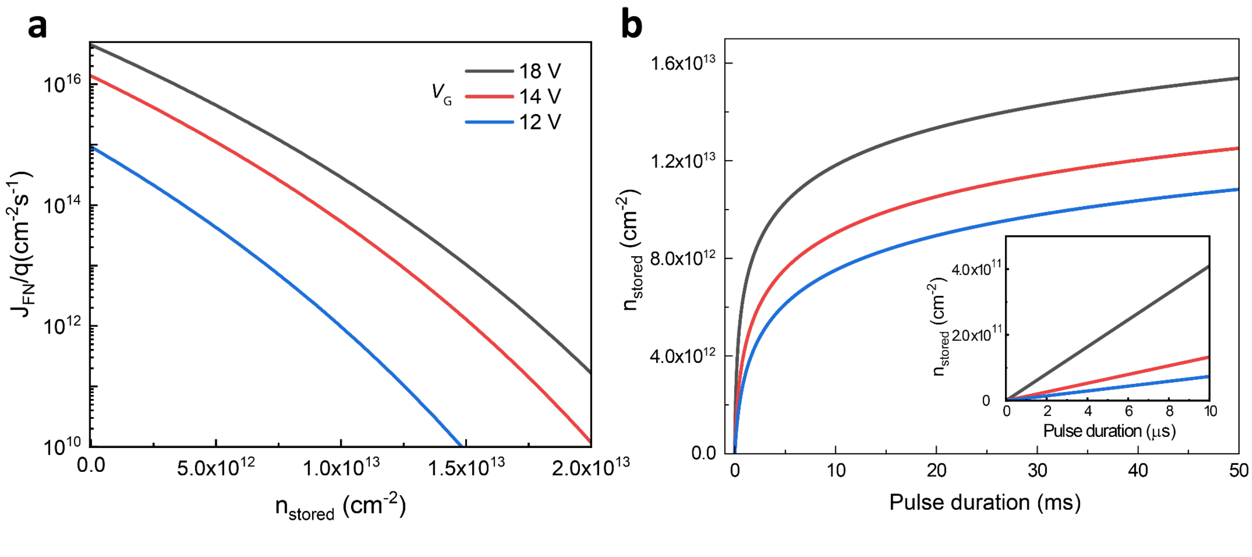

Supplementary Note 6. For optical programming, we used optical pulses of ms duration in our experiment. But based on the theoretical analysis, the programming speed can be increased with ns~μs optical pulses, depending on the pulse intensity and photon energy. Mechanisms of de-trapping of the stored charges in the HfO_2 layer include field-assisted tunneling, thermally excited charge tunneling, and optically excited charge tunneling as indicated in equation (7). Without the gate voltage, the tunneling coefficients for the first two mechanisms are negligible compared to the optically excited tunneling coefficient as shown in Supplementary Figure 8a. Because the trapped charges have to overcome the large tunneling barrier with Φ_B', the charge tunneling process can be activated with optical illumination, which reduces the effective barrier to $\Phi_B' - \hbar\omega$. The optically assisted tunneling process can be described as:

$$
\begin{aligned}
-\frac{\mathrm{d}n_{stored}}{dt} &\propto TC\left(\xi_{opt}, E_{built}(n_{stored})\right) * n_{opt} \\
&= TC\left(\xi_{opt}, E_{built}(n_{stored})\right) * \left(G(P_{opt}) * t\right) \qquad (7)
\end{aligned}
$$

where G is charge generation rate due to the optical illumination, which is assumed to be proportional to the optical power P_{opt}. To solve this nonlinear differential equation, we assume the conductance of the bP channel is in the range of 30 – 80 μS, which corresponds to the stored charge density of 4 – 12 $\times 10^{12}$ cm^{-2}, as discussed in S.I. Note 3. Also, we approximated the analytical function of *TC* with an exponential function and only considered the trap site with 1.25 eV below the conduction band, where the largest density of oxygen vacancies in HfO_2 exists as reported with optical absorption spectra[12].

The resulting trapped charge density n_{stored} versus illumination time is plotted in Supplementary Figure 8b. The operation speed can be further reduced to the ns regime with higher photon energy and higher optical power. Considering our experimental conditions: the optical power on the bP-PPT device is 12 μW, pulse width is varied from 1 ms to 200 ms, and photon energy is 1.6 eV (780 nm). We use equation (7) to calculate the optical pulse energy required to change the device from state #(n-1) to state #n, which is plotted in Supplementary Figure 8c. The model shows a good agreement with the experimental results in Supplementary Figure 5a.

Supplementary Figure 8. The optical programming speed. (a) Tunneling coefficients depending on the stored charge density in the storage layer. (b) The conductance of bP-channel changes depending on the optical power and illumination time. (c) The calculated and experimental optical energy depending on the pulse number n, that changes the device from state #(n-1) to #n.

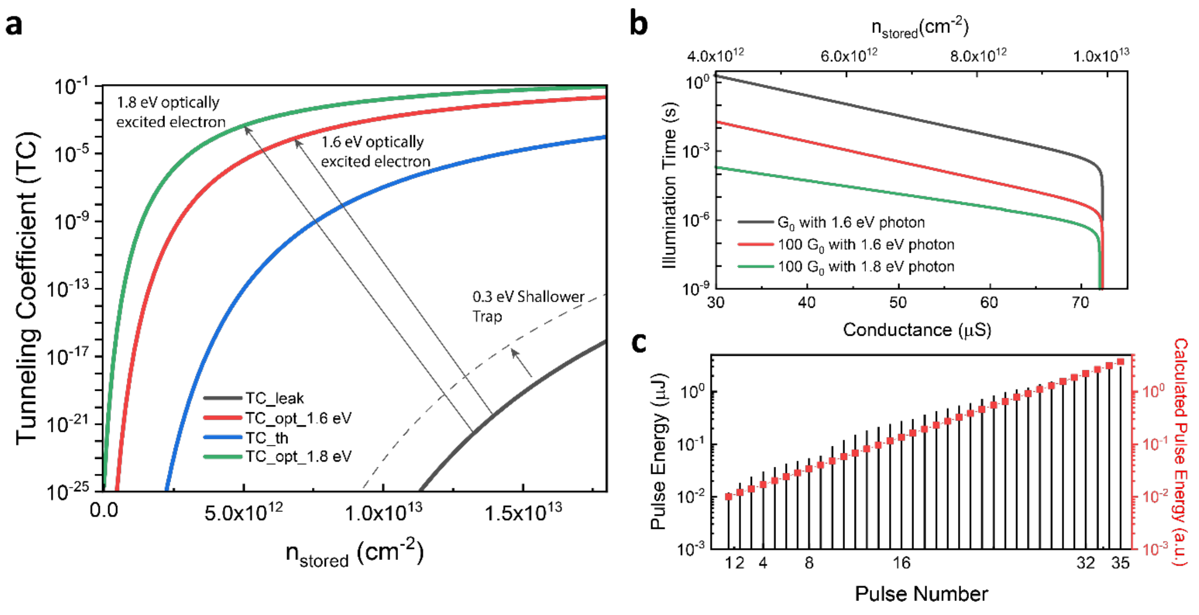

Supplementary Figure 9. Optical Microscope image of bP flake for (a) as transferred and (b) after patterning to the bP-PPT device array. All 12 devices are fabricated from the flat region of the bP flake as outlined with red-dashed line to minimize the device-to-device variation. Each device has source and drain contacts and an ITO top gate electrode. Inset: the thickness of bP by AFM along the white dashed line. Scale bar: 10 μm.

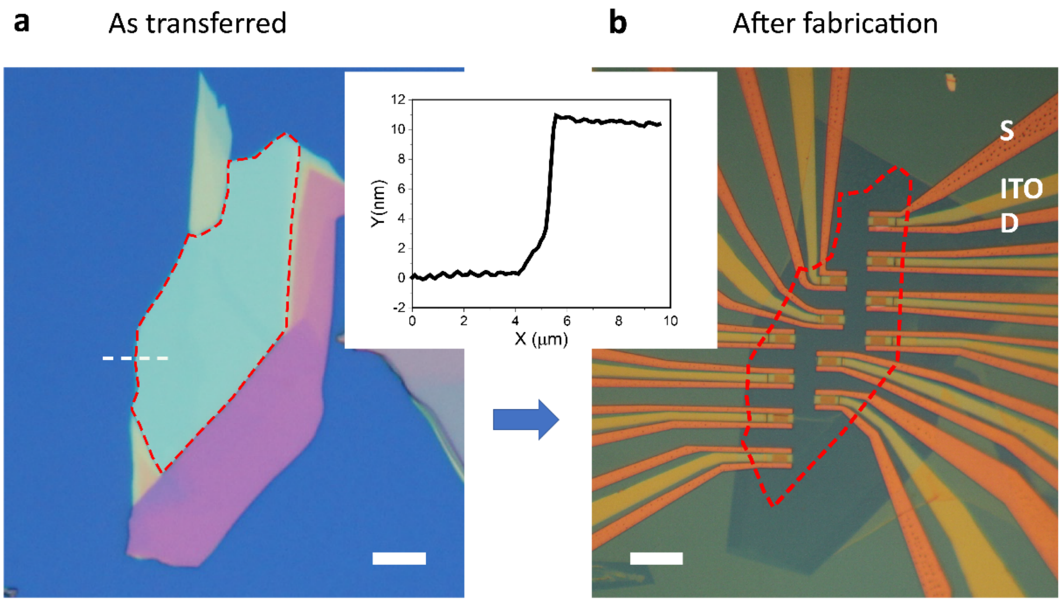

Supplementary Note 7. Characterization of bP degradation during the fabrication process is performed by repeating the bP patterning process (e-beam lithography, ICP etch, resist removal). The optical microscopic images and AFM measurements of a bP flake as transferred and after etching are shown in Supplementary Figure 10a. The freshly exfoliated bP flake with a flat surface with 17-nm thickness is preserved in its optical contrast, surface smoothness, and thickness after the ICP etching. The thickness of the bP rectangles can be estimated considering the etched thickness of SiO_2 substrate as shown in the right column of Supplementary Figure 10a. Additionally, the Raman spectroscopy of the bP rectangle in Supplementary Figure 10b shows no signs of P_xO_y or H_xPO_y, at around Raman signal from 800 to 1000 cm^{-1}, revealing that oxidation and degradation of bP during the fabrication process is negligible[15].

Supplementary Figure 10. Degradation test of the bP flake upon the device fabrication process. (a) Optical images and AFM measurements for flat surface and preserved thickness of a bP flake upon ICP etching into two rectangles. The step height measurements are performed along the white dashed lines. The thickness of the bP flake is maintained as 17 nm. (b) Raman spectroscopy for ICP etched bP rectangle. It shows no indication of P_xO_y or H_xPO_y formed during the fabrication process.

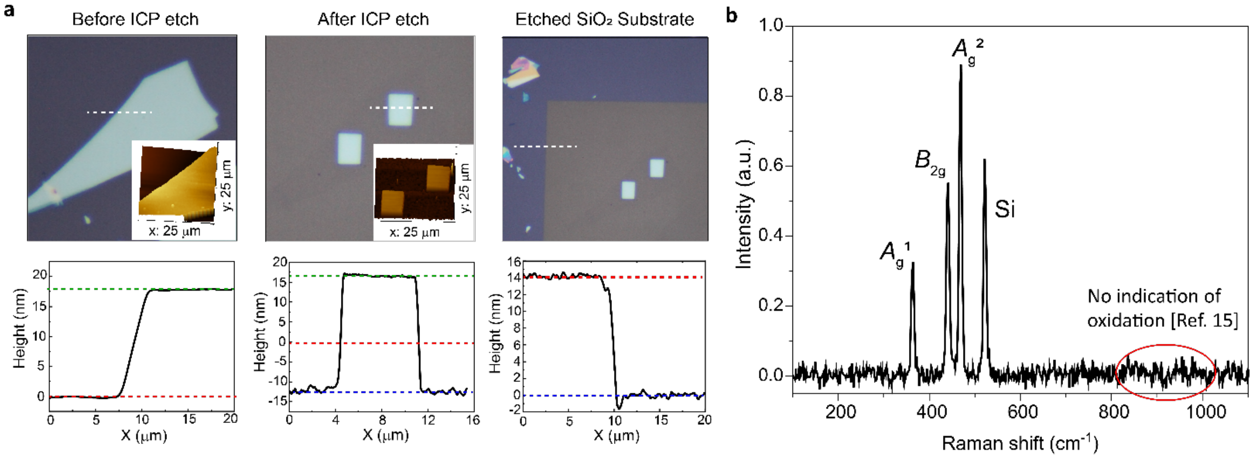

Supplementary Figure 11. Estimation of lifetime of bP-PPT device. (a) I_{ds}-V_G measurement and (b) time traced retention of two states of a bP-PPT device over 3 months. Both show the good consistency of gate modulation, memory windows, on-off ratio, and retention time even after the 3 months.

Note: Considering our device geometry, we have 35 nm AHA oxide (Al_2O_3/ HfO_2/Al_2O_3) layers grown by atomic layer deposition (ALD) which can effectively passivate the bP and improve the stability of the bP devices. It is previously demonstrated that the 20 nm ALD oxide (Al_2O_3) can conformally coat the device and protect the thin bP flake from degradation for more than 90 days[16].

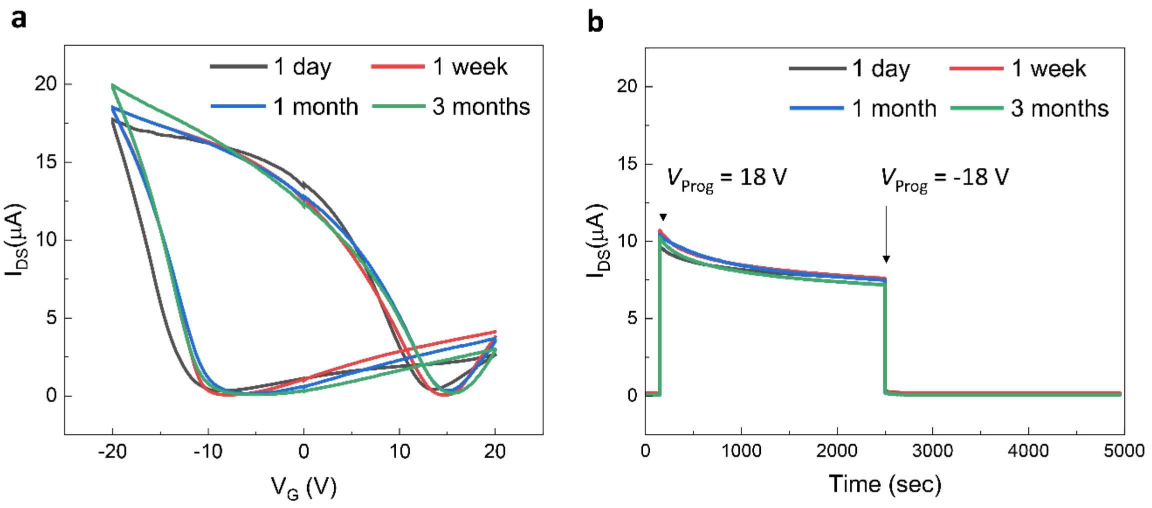

Supplementary Note 8. The 36-level-high-precision programmability of the bP-PPT device is used to compute the convolutional layer with two kernels of the CNN explained in Fig. 4a. The 9 devices of bP-PPT which performs most closely were selected to exhibit the 3 × 3 kernels with the element indicated in Fig. 4b in the main text. The target conductance levels are decided by the following equation:

$$g_{i,j}^{target} = g_{i,j}^{+} - g_{ref,j} = k\left(\left|w_{i,j}^{+}\right| - \left|w_{i,j}^{-}\right|\right) = k\, w_{i,j}^{target}$$

, where $g_{i,j}^{target}$ indicates the target conductance with both positive and negative values. $g_{i,j}^{+}$ and $g_{ref,j}$ indicate the device conductance at a pixel in row i and column j, and at reference pixel of column j, respectively, with only positive values. Similarly, $w_{i,j}^{target}$ is the target weight which is obtained from an offline CNN and k is the coefficient to relate the weights and the conductance. $w_{i,j}^{\pm}$ represent the target weight in its positive and negative values. Since the 9 pixels are all used for the convolution, the reference is done by reprogramming the bP-PPT devices to the reference weights. The product of input voltage vector $\boldsymbol{v}\ (m \times 1)$ and the weight conductance matrix $g(m \times n)$ produces the output current vector $\boldsymbol{i}\ (n \times 1)$. In our case, n=1 and m=9 for each kernel. At each time frame of the computation, a convolution value of a pixel among 26 × 26 of the convolved image is read out as a total current, whose time trace is shown in Supplementary Figure 12. The two 3 × 3 kernels are discretized to take the nearest values of the ideally trained kernel from offline CNN, as tabled in Supplementary Table 2.

Supplementary Figure 12. Raw data of the time trace of the convolutional result for the symbol "0".

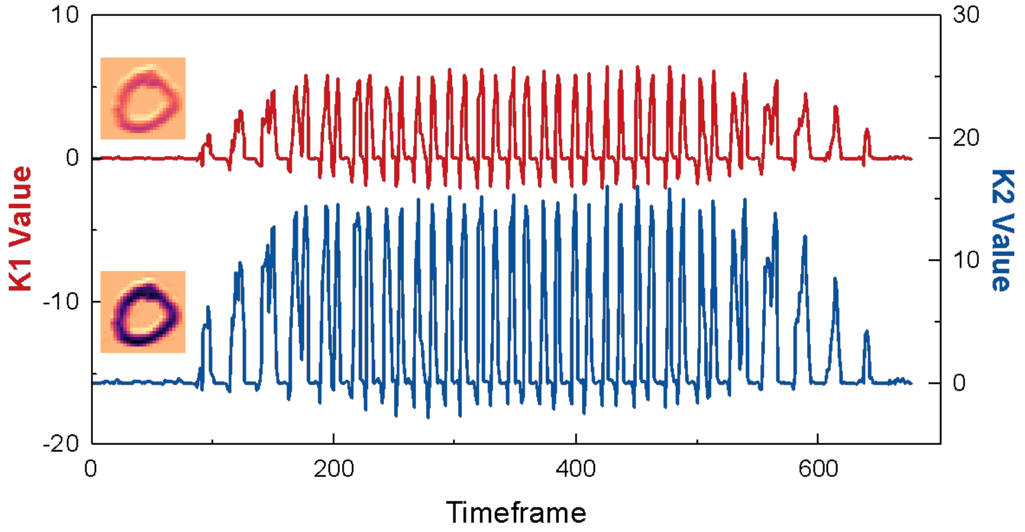

Supplementary Table 2. Discrete Kernel values compared to the kernel values of ideally trained CNN for two convolution kernels **K1** and **K2** (Fig. 4b). The bP-PPT devices are set to the state numbers from #0 to #35 and reference numbers to cover the weight values spanning from positive to negative values.

K1								
**Device state no. (#i - #ref)**			**Ideally trained Kernel**			**Discrete Kernel**		
**34 (#34 - #0)**	**11 (#21 - #10)**	**-17 (#3 - #20)**	1.9539	0.6355	-0.9577	**1.94**	**0.63**	**-0.97**
**35 (#35 - #0)**	**9 (#19 - #10)**	**-15 (#5 - #20)**	2.0035	0.4924	-0.8589	**2.00**	**0.51**	**-0.86**
**28 (#28 - #0)**	**3 (#13 - #10)**	**-13 (#7 - #20)**	1.6262	0.1575	-0.7946	**1.60**	**0.17**	**-0.74**
**K2**								
**Device state no. (# i - # ref)**			**Ideally trained Kernel**			**Discrete Kernel**		
**25 (#25 - #0)**	**11 (#21 - #10)**	**-6 (#14 - #20)**	3.477	1.5833	-0.773	**3.43**	**1.51**	**-0.82**
**28 (#28 - #0)**	**12 (#22 - #10)**	**-8 (#12 - #20)**	3.7587	1.6116	-1.091	**3.84**	**1.64**	**-1.09**
**35 (#35 - #0)**	**13 (#23 - #10)**	**-13 (#7 - #20)**	4.7789	1.7567	-1.7929	**4.80**	**1.78**	**-1.78**

Supplementary Figure 13. Evaluation of the edge detection result by comparing the measured values of each pixel to the simulated results for all three pictures (MNIST handwritten digits, a husky dog, and a cameraman). (a) Experimental output photocurrent versus the simulated result, which shows linear correlation. (b) The correlation coefficients between the experimentally processed images and simulated results. All three pictures show the correlation coefficient over 92%.

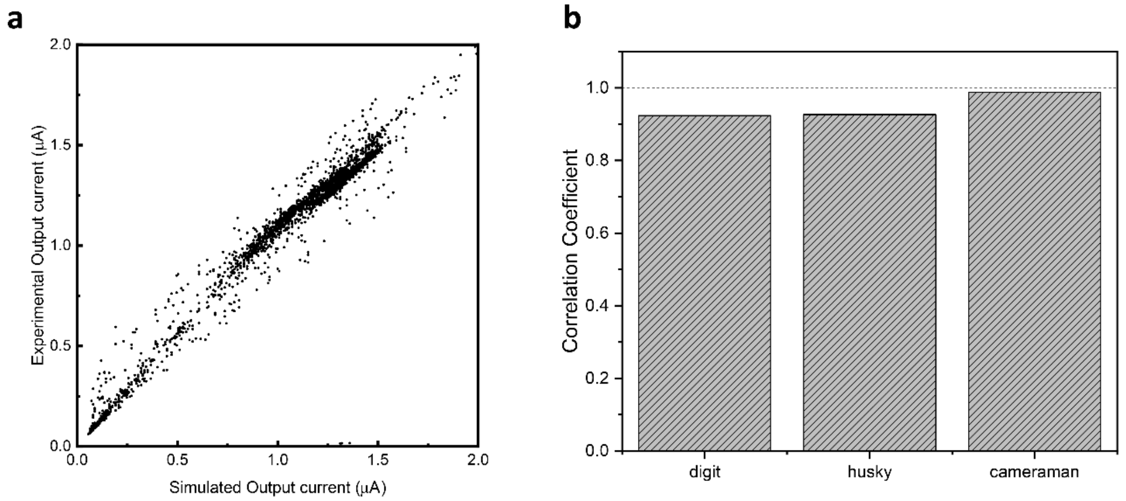

Experimental set up.

The bP-PPT devices were wire-bonded to the 64-pin chip holder as shown in Supplementary Figure 14. As for the electrical measurements of the bP devices, each device was wired to 3 pins for the individual connection to the gate, source, and drain. Since we have 12 bP-PPT devices, we can simultaneously measure the conductance or photocurrent of several devices using SMU modules. For the optical input, both 780 nm and telecom-band laser beams were aligned and focused onto one device. We programmed the devices using a 780 nm laser diode (LP785SF20, Thorlabs) with tunable output power and pulse width. The optical images were input to the devices by modulating the intensity of the telecom laser (TSL-210, Santec Corporation) using a variable optical attenuator (EVOA1550A, Thorlabs), and detected by measuring the photocurrents of the bP-PPT devices.

The optical programming can be done parallelly with a spatial light modulator. For the future packaging of the edge computing sensor, we can connect the preprocessing devices to a local data acquisition system[17] and the output from the circuit can be feedback to the postprocessing bP-PPT devices working as a deeper layer of the network.

Moreover, to characterize the programmable responsivity in the broadband infrared spectrum, a tunable infrared OPO laser (Firefly, M Squared) has been used with its signal beam for the wavelength 1.5-1.8 μm and with its idler beam for the wavelength 2.6-3.1 μm. The signal and idler beams are focused onto the bP-PPT device with the CaF_2 lens.

Supplementary Figure 14. Measurement scheme for the bP-PPT array.

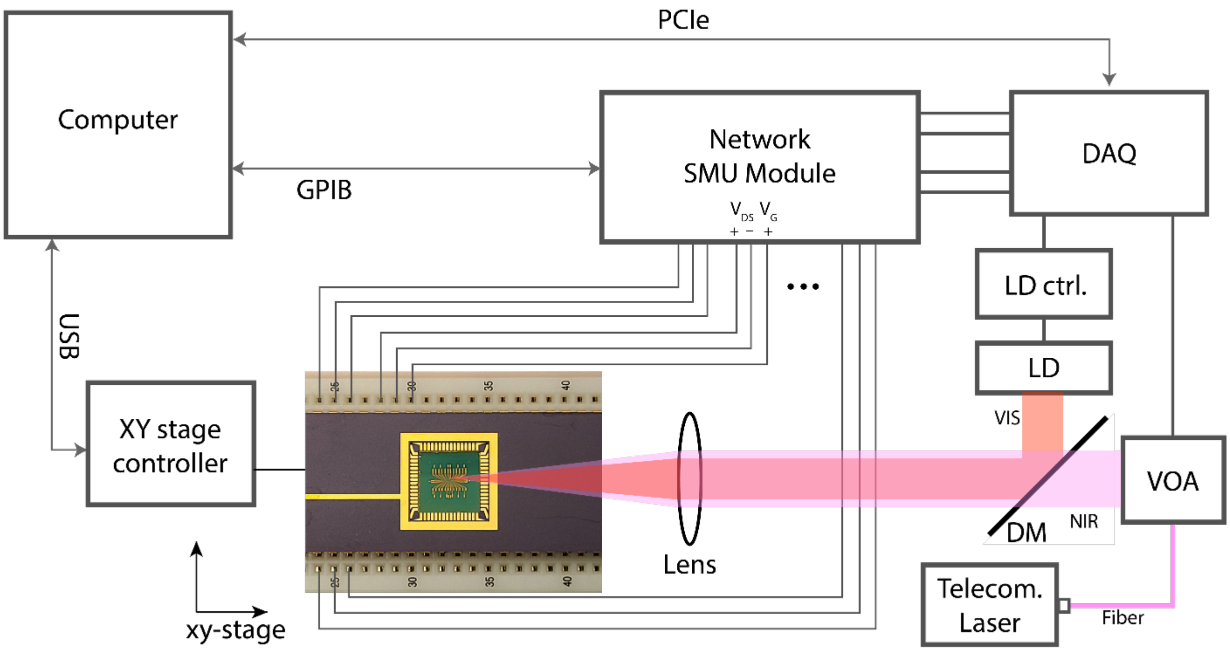

Methods

bP-PPT Device Fabrication

Few-layer black phosphorus (bP) with 11 nm thickness is mechanically exfoliated from a bulk crystal (HQ Graphene) and dry transferred with a PDMS stamp onto 300-nm-thick thermally grown SiO_2/Si substrate. The thickness of the bP flake is identified by AFM (Bruker Dimensions Icon). The bP flake with the lateral size of 20 μm × 30 μm was patterned into an array of 4 × 3 pixels with each pixel size of 3 μm × 4 μm (Fig. 1c) by Ebeam lithography and SF_6 inductively coupled plasma (ICP) etching with the ZEP 520A as a protective mask. Source and drain contacts (5 nm /25 nm, Ni/Au) were patterned by standard E-beam lithography (JEOL- JBX6300FS) and then deposited by the E-beam evaporation. The exfoliation of bP, the thickness measurement, and the lift-off process of deposited metal were all performed in the Ar-filled glovebox with oxygen and water molecule <0.1 ppm to avoid the degradation of the bP flake by minimizing the exposure to moisture and oxygen. Subsequently, the gate dielectric stack of Al_2O_3/HfO_2/Al_2O_3 (AHA) was grown over the array by an atomic layer deposition (ALD) system. The 6-nm-thick tunneling layer of Al_2O_3 was grown on bP at 150 ˚C by thermal ALD, and the 7-nm-thick storage layer of HfO_2 was then grown at 290 ˚C by plasma-enhanced ALD, followed by the 20-nm-thick blocking layer of Al_2O_3. On top of the gate stack, Indium-tin-oxide (ITO), a transparent gate electrode, was patterned and deposited by E-beam lithography and a pulsed sputtering system (Evatec LLS EVO), respectively.

References

1. Ahmed, T., Tahir, M., Low, M.X., Ren, Y., Tawfik, S.A., Mayes, E.L.H., Kuriakose, S., Nawaz, S., Spencer, M.J.S., Chen, H., Bhaskaran, M., Sriram, S. & Waila, S. Fully Light-Controlled Memory and Neuromorphic Computation in Layered Black Phosphorus. *Adv. Mater.* **33**, 2004207 (2021).

2. Seo, S., Jo, S.-H., Kim, S., Shim, J., Oh, S., Kim, J.-H., Heo, K., Choi, J.-W., Choi, C., Oh, S., Kuzum, D., Wong, H.-S. P. & Park, J.-H. Artificial optic-neural synapse for colored and color-mixed pattern recognition. *Nat. Commun.* **9**, 5106 (2018).

3. Jang, H., Liu, C., Hinton, H., Lee, M.-H., Kim, H., Seol, M., Shin, H.-J., Park, S. & Ham, D. An Atomically Thin Optoelectronic Machine Vision Processor. *Adv. Mater.* **32**, 2002431 (2020).

4. Feng, Q., Yan, F., Luo, W. & Wang, K. Charge trap memory based on few-layer black phosphorus. *Nanoscale* **8**, 2686-2692 (2016)

5. Lee, D., Choi, Y., Hwang, E., Kang, M. S., Lee, S., & Cho, J. H. Black phosphorus nonvolatile transistor memory. *Nanoscale* **8**, 9107–9112 (2016).

6. Liu, L., Liu, C., Jiang, L., Li, J., Ding, Y., Wang, S., Jiang, Y.G., Sun, Y.B., Wang, J., Chen, S., Zhang, D.W. ... & Zhou, P. Ultrafast non-volatile flash memory based on van der Waals heterostructures. *Nat. Nanotechnol.* **16**, 874–881 (2021).

7. Wu, L. Wang, A., Shi, J., Yan, J., Zhou, Z., Bian, C., ... & Gao, H. J. Atomically sharp interface enabled ultrahigh-speed non-volatile memory devices. *Nat. Nanotechnol.* **16**, 882–887 (2021).

8. Mennel, L., Symonowicz, J., Wachter, S., Polyushkin, D. K., Molina-Mendoza, A. J., & Mueller, T. Ultrafast machine vision with 2D material neural network image sensors. *Nature* **579**, 62–66 (2020).

9. Migliato Marega, G., Zhao, Y., Avsar, A., Wang, Z., Tripathi, M., Radenovic, A., & Kis, A. Logic-in-memory based on an atomically thin semiconductor. *Nature* **587**, 72–77 (2020).

10. Wang, S. T. Charge Retention of Floating-Gate Transistors Under Applied Bias Conditions. *IEEE Trans. Electron Devices* **27**, 297–299 (1980).

11. Youngblood, N., Chen, C., Koester, S. J. & Li, M. Waveguide-integrated black phosphorus photodetector with high responsivity and low dark current. *Nat. Photonics* **9**, 247–252 (2015).

12. Gritsenko, V. A., Perevalov, T. V. & Islamov, D. R. Electronic properties of hafnium oxide: A contribution from defects and traps. *Phys. Rep.* **613**, 1–20 (2016).

13. Chang, C. H. & Hwu, J. G. Trapping characteristics of Al2O3/HfO2/SiO2 stack structure prepared by low temperature in situ oxidation in dc sputtering. *J. Appl. Phys.* **105**, 094103 (2009).

14. Tsu, R. & Esaki, L. Tunneling in a finite superlattice. *Appl. Phys. Lett.* **22**, 562 (2003).

15. Naqvi, B. A., Shehzad, M. A., Cha, J., Min, K. A., Khan, M. F., Hussain, S., ... & Jung, J. Visualizing Degradation of Black Phosphorus Using Liquid Crystals. *Sci. Reports* **8**, 1–8 (2018).

16. Gamage, S., Li, Z., Yakovlev, V. S., Lewis, C., Wang, H., Cronin, S. B., & Abate, Y. Nanoscopy of Black Phosphorus Degradation. *Adv. Mater. Interfaces* **3**, 1600121 (2016).

17. Choi, C., Leem, J., Kim, M. S., Taqieddin, A., Cho, C., Cho, K. W. & Kim, D. H. Curved neuromorphic image sensor array using a MoS2-organic heterostructure inspired by the human visual recognition system. *Nat. Commun.* **11**, 1–9 (2020).
